# Administering the MADRS by telephone or face-to-face: a validity study

**DOI:** 10.1186/1744-859X-5-3

**Published:** 2006-03-22

**Authors:** Marleen LM Hermens, Herman J Adèr, Hein PJ van Hout, Berend Terluin, Richard van Dyck, Marten de Haan

**Affiliations:** 1 Department of General Practice, Institute for Research in Extramural Medicine, VU University Medical Center, Amsterdam, The Netherlands; 2 Department of Clinical Epidemiology and Biostatistics, VU University Medical Center, Amsterdam, The Netherlands; 3 Department of Psychiatry, Institute for Research in Extramural Medicine, VU University Medical Center, Amsterdam, The Netherlands

## Abstract

**Background:**

The Montgomery Åsberg Depression Rating Scale (MADRS) is a frequently used observer-rated depression scale. In the present study, a telephonic rating was compared with a face-to-face rating in 66 primary care patients with minor or mild-major depression. The aim of the present study was to assess the validity of the administration by telephone. Additional objective was to study the validity of the first item, 'apparent sadness', the only item purely based on observation.

**Methods:**

The present study was a validity study. During an in-person interview at the patient's home a trained interviewer administered the MADRS. A few days later the MADRS was administered again, but now by telephone and by a different interviewer. The validity of the telephone rating was calculated through the appropriate intraclass correlation coefficient (ICC).

**Results:**

Mean total score on the in-person administration was 24.0 (SD = 11.1), and on the telephone administration 23.5 (SD = 10.4). The ICC for the full scale was 0.65. Homogeneity analysis showed that the observation item 'apparent sadness' fitted well into the scale.

**Conclusion:**

The full MADRS, including the observation item 'apparent sadness', can be administered reliably by telephone.

## Introduction

The Montgomery Åsberg Depression Rating Scale (MADRS) is one of the most frequently used and validated observer-rated depression scales. The scale was developed more than 20 years ago but is still favorite among researchers to measure the severity of depressive disorders and the changes of depressive symptoms during therapy [1]. Until now, the MADRS was only used in an in-person situation with the depressed patient. It is not clear whether the MADRS can be reliably administered by telephone.

The fact that patient and interviewer have to meet face-to-face makes the MADRS rather cost- and time-consuming. Almost a decade ago a self-rating version of the MADRS, the MADRS-S, was published. It was claimed to be equivalent to the Beck Depression Inventory (BDI), also a self-rating instrument for depression [2]. The scales were highly intercorrelated (r = 0.869). The BDI is the most widely used self-rating depression scale [3]. While the self-rating version of the MADRS can make a contribution in reducing costs, it suffers from at least two limitations. The first limitation is that there are no observers involved. Clinicians may prefer an observer-rated scale for different reasons, for example because self-perception of patients with severe depressions can be distorted [4], or items can be misunderstood. Second, one item of the original MADRS, 'apparent sadness', is based exclusively on observation of the interviewer and could therefore not be included. Thus, the self-rating version consists of nine instead of 10 items.

We took another approach to solve the problem: administering the MADRS by telephone. Telephone administration may have several advantages. It (a) can include all original items, (b) preserves the characteristic of a clinical interview, and (c) is less costly and time-consuming than in-person administration. Previous studies have examined the comparability of face-to-face and telephone-administered interviews for obtaining data on health status or psychiatric symptoms [5-8]. These studies indicate that telephone-administered interviews are at least as valid as data obtained from face-to-face interviews.

The objective of this study was to assess the validity of the telephonic rating of the full scale by comparing it with the rating obtained during an in-person interview. More precisely, we wanted to assess the convergent validity, i.e. to establish whether the telephonic rating measures the same construct and returns similar results as the face-to-face rating. Additional objective was to study the validity of the observation item, 'apparent sadness'.

## Methods

### Research design

The present study was a validity study among primary care patients suffering from minor or mild-major depression, based on criteria of the Diagnostic and statistical manual of mental disorders, 4th edition (DSM-IV) [9]. The MADRS was first administered in-person by a trained interviewer who discussed each item with the patient. A different interviewer, blind to the findings of the first interview, administered the MADRS within a few days interval by telephone. The investigation was carried out in accordance with the latest version of the Declaration of Helsinki [10] and an ethical committee reviewed and approved the study design.

### Patients

This study was part of a trial to evaluate the treatment of minor and mild-major depression by general practitioners (GPs). The study was conducted in 2002 and 2003 in the Netherlands. Patients were included if the GP assessed 3–6 out of 9 DSM-IV symptoms of depression (including at least one of the core symptoms 'sadness' or 'loss of pleasure'). The symptoms had to be present for at least 2 weeks, causing occupational or social impairment. Largely in accordance with DSM-IV [9], we defined mild-major depression as a depressive disorder with 5–6 symptoms. In accordance with the Dutch guideline on depression [11], issued by the Dutch College of General Practitioners, but not entirely in accordance to the DSM-IV, we defined minor depression as a depressive disorder with 3–4 symptoms. Patients were excluded if they were 17 years or younger, pregnant or breast-feeding, already receiving anti-depressant medication or specialized treatment, having an addiction to alcohol or drugs, experiencing bereavement, or if psychotic features accompanied the depressive symptoms. Additionally, there were some extra exclusion criteria concerning the practical ability to participate in the study. Patients were excluded if they were not able to complete questionnaires due to language difficulties, illiteracy or cognitive decline or if they did not have a telephone.

As a check of the GP's diagnoses, but without consequences for the inclusion in the study, standardized psychiatric diagnoses were obtained with the Composite International Diagnostic Interview (CIDI) [12] during the baseline interview.

Every consecutive patient entering the study was asked to participate in the present validity study. We aimed to include a total of 70 patients. This number was considered sufficient to obtain reliable estimates of the variance components that were needed [13].

### The MADRS

The MADRS is a 10-item rating scale to assess the severity of depressive symptoms within the last 7 days. The items were taken from the 65-item Comprehensive Psychopathological Rating Scale (CPRS) and were selected because of their sensitivity to change [14,15]. The 10 selected items are rated on a scale of 0-6 with anchors at 2-point intervals. The interviewer is encouraged to use his or her observations of the patient's mental status as an additional source of information. Total scores on the MADRS range from 0 to 60 [1]. For the present study, the Dutch translation of the MADRS was used. It has been shown to have high inter-rater reliability (spearman r = 0.94) and good concurrent validity (r with HAM-D between 0.83 and 0.94) [4].

As mentioned in the introduction, the first item of the MADRS, 'apparent sadness', is based exclusively on the observation of the interviewer, unlike the other 9 items. The interviewer assesses the level of sadness the patient exhibits during the interview by being attentive to non-verbal signals like speech, facial expressions and posture. However, during the telephone interview no visual signs can be observed. To compensate for this, interviewers were instructed to be attentive to all verbal signs, like tone of voice, rhythm, pace of talking, and other sounds during the interview, like sighing or crying, to assess the level of sadness the patient was experiencing.

### Procedure

When the GP saw an eligible patient with depressive symptoms, the research assistant at the VU University Medical Center in Amsterdam was notified. Then, one of the interviewers contacted the patient and made an appointment for an in-person interview at the patient's home within two weeks. During this home visit the interviewer administered the MADRS, the CIDI and other scales and questionnaires. After this, the interviewer explained the aim of the present validity study. If the patient was willing to participate, the research assistant was notified, who arranged for a different interviewer to contact the patient as soon as possible (0 to 4 days after the initial interview) to administer the MADRS by telephone.

The MADRS was administered in the middle of the interview. This may have helped to prevent a primacy effect, a memory effect within patients that may occur if the MADRS would have been administered at the beginning, or a recency effect, if the MADRS would have been administered at the end [16].

Robins [17] has described desirable characteristics of studies of agreement between psychiatric measures: (1) the order of administration should be reversed for a random sample of the participants to compensate for any sequence effects; (2) the time interval between administrations should be minimized and recency effects should be determined; and (3) the measures should be administered to the same sample rather than each measure administered to a different random subsample. Our study design addressed all but the first of these recommendations. The reason for this assessment order (first face-to-face, then telephone) was of a practical nature: the present study was part of a larger trial which left no room for changes in procedures.

In short, the MADRS was administered twice to the same participants by two different interviewers, first face-to-face, then by telephone. During the interval between administrations, the two interviewers had no contact and no information about the patient was shared between them.

### Interviewers

Nine well-trained lay interviewers assessed the patients. Experts at the Psychiatric Clinic of the VU University Medical Center in Amsterdam, the Netherlands, trained the interviewers in administering the MADRS. Interviewers each performed both in-person and telephone interviews.

### Statistical analyses

Variance component analysis was used to partition the total variability into components of variation due to Patients, Assessment Mode (face-to-face or telephonic), and Measurement error [18]. The first research aim was concerned with the convergent validity of the telephonic versus the in-person assessment of the full scale. For the second research aim, concerning item 1, 'apparent sadness', the variance component analysis of item 2 to 10 was compared with the analysis of full scale on both assessments. We also fitted a model in which the two aims were combined. All three models included a covariate for the number of days between the ratings to compensate for a possible memory effect.

Results were obtained over the full scale and over item 2 to 10 as the total variability and the percentage of the total variability attributable to each variance component. The validity of the telephonic rating mode was calculated from the variance (var) components through the appropriate intraclass correlation coefficient (ICC) according to the following formula [19-21]:



The ICC is a measure for the agreement between the modes of assessment. The closer the ICC is to 1, the better the agreement. An ICC <0.30 signifies low agreement, 0.30–0.60 moderate agreement, 0.60–0.80 acceptable agreement, and >0.80 means high agreement. In addition, homogeneity analyses on the MADRS scale, reported as Cronbach's alpha, for both the in-person and the telephone administration were carried out to see if item 1, "apparent sadness", fitted well into the scale.

Differences between the total scores on the MADRS, administered at both interviews, are depicted in a Bland-Altman plot. The Bland-Altman plot is useful in showing the amount of agreement between the two modes of administration. The 'limits of agreement' are calculated (mean difference ± 2*SD) defining the range that contains 95% of all differences [19,22,23]. Statistical calculations were performed using SPSS 11.0.

Finally, confirmatory factor analysis (CFA, using the software program EQS) was used to calculate the parameters of the observation item and the scales constituted by the rest of the items in the telephonic and face-to-face administration. This analysis was used to demonstrate congenericity [24]. Congenericity means that the same trait was measured, except for errors of measurement. The test of Wilks [25] was used to demonstrate parallelism of the two administrations of the full scale. Parallel scales are scales that measure the same construct and have equal means and equal variances.

## Results

### Descriptive statistics

Seventy patients consented to participate in the validity study (82% of 85 consecutive patients asked). The main reason for not wanting to participate was the patients' inability to cooperate due to lack of time or opportunity. Data from four patients were excluded from the analysis due to procedural errors. Therefore, the statistical analyses were based on data from 66 patients.

The sample consisted of 20 males and 46 females. Mean age was 44 (SD = 17, range 19–79). The mean number of days between the two ratings was 3.1 (SD = 2.0, range 0–9). Mean total number of depressive symptoms according to the diagnosis of the GP was 5.2 (SD = 0.9, range 3.0–6.0). CIDI diagnoses of 65 patients were obtained. Thirty-nine patients (60%) were diagnosed with a current major depressive disorder; 13 had a mild, 12 had a moderate, and 14 had a severe major depressive disorder. Ten patients (15%) suffered from (co-morbid) dysthymia. Mean total score on in-person administration of the MADRS was 24.0 (SD = 11.1, range 0.0–54.0). Mean score of the telephone administration was 23.5 (SD = 10.4, range 1.0–54.4). The mean difference between the telephone and in-person ratings was -0.5 (SD = 6.9, range -19.0–22.0).

### Results concerning the full scale

Variance component analysis showed that Measurement Error determined most of the variance (35.2%), whereas 29.8% could be ascribed to between-patient variability. Some variance (5.7%) was determined by the Assessment Mode (the way the MADRS was administered). Based on the variance component analysis the calculated ICC was 0.65. Results of the variance component analysis are shown in Table 1.

**Table 1:** Results of the variance component analysis for the full scale and for item 2 to 10

	Variance components	Percentages of total (%)	Estimates of the variance components
Full scale	Patients	29.8	0.824
	Assessment Mode^a^	5.7	0.157
	Measurement error^b^	35.2	0.973
	Residual error	29.4	0.814
Item 2 to 10	Patients	28.7	0.808
	Assessment Mode	5.5	0.154
	Measurement error	38.0	1.074
	Residual error	27.8	0.783
Combined model	Patients	34.5	0.958
	Test length by Mode	0.8	0.02
	Measurement + Residual error	64.8	1.80

^a ^Assessment Mode: face-to-face or telephonic

^b ^Measurement error was assessed by the Patient * Item terms

**Figure 1:**
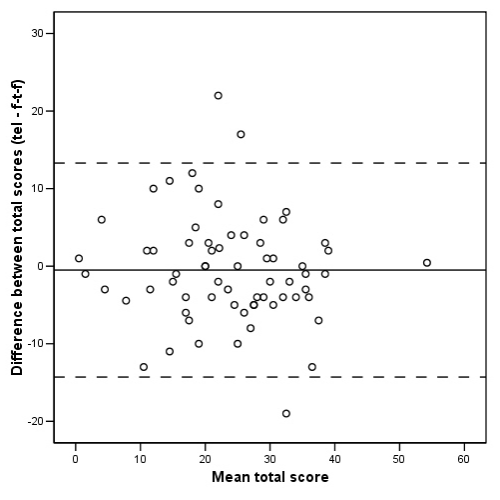
**Bland-Altman plot of the difference in total MADRS scores against the mean of the total scores at both interviews**. The straight line represents the mean difference; the dotted lines represent the 'limits of agreement' (mean difference ± 2 SD difference)

Furthermore, Figure 1 depicts a Bland-Altman plot of the mean difference in total scores against the mean of the total scores at both interviews. The mean difference was -0.5 (95% CI -2.2 to 1.2; p = 0.56). The limits of agreement were -14.3 and 13.3. This indicates that the second MADRS score was with 95 percent certainty less than 13.8 points away from the first MADRS score. The variation between the two scores was largely due to the moderate measurement precision of the MADRS itself, irrespective of the mode of administration.

### Results on item 1, 'apparent sadness'

A comparison of the variance component analysis of item 2 to 10 and the full scale showed that the variance determined by the components of item 2 to 10 was in line with the full scale. Accordingly, the ICC of item 2 to 10 was comparable with the ICC for the full scale: based on the variance component analysis, the calculated ICC for the total score of item 2 to 10 was 0.66 (for the full scale it was 0.65, as mentioned in the previous section). Since item 1 does not seem to have much influence on the scale, the full scale can be maintained. Results of the variance component analyses for item 2 to 10 and for the full scale are shown in Table 1.

### Results for a combined model

In a combined model, in which both Scale Length and Assessment Mode were included, 34.5% of the variance could be ascribed to Patients, while 0.8% of the variance was ascribed to the interaction between Scale Length and Assessment Mode. Other interaction terms and main effects in the model were negligible (see Table 1).

### Internal consistency

Homogeneity analysis showed that both administration modes lead to homogeneous scales. Moreover, it showed that the internal consistency of the telephonic as well as the face-to-face scale did not change when item 1 was left out. Cronbach's alfa of the in-person administration of the full scale was 0.85; without item 1 it was 0.84. Cronbach's alfa of the telephone administration of the full the MADRS was 0.81; without item 1 it was 0.78. These results showed that differences in internal consistency, both with and without item 1, were only marginal.

### Congenericity and parallelism

The two-factor confirmatory factor analysis using structural equation model with factors 'By Telephone' (T) and 'Face-to-Face' (F) had a comparative fit index (CFI) of 0.767, while the β-coefficients were as follows: (I_1,F_, F_9_) = 0.933; (I_1,T_, T_9_) = 0.944. The correlation between F_10 _and T_10 _was 0.836, which gave (moderate) support to the hypothesis of congenericity. The test of Wilks [25] was not significant, neither for the 10 item scales (χ^2 ^df2 (F,T) = 5.08; p > 0.05) nor for the 9 item scales (χ^2 ^df2 (F,T) = 5.06; p > 0.05). Therefore the hypothesis of parallelism could not be rejected.

## Discussion

Regarding the main research aim, concerning the validity of the telephone rating of the MADRS, we can conclude the following. The acceptable agreement between the telephone and the face-to-face assessment suggested that the telephone rating is valid. Furthermore, parallelism was demonstrated between the two scales. The results further show that the mode of administration determined some, but not much, of the variance. In addition, the mean difference between both administration modes proved to be small. The Bland-Altman plot shows that there was much variation, and because not much variance was determined by the administration mode, this suggests a moderate measurement precision of the MADRS itself. This interpretation was also supported by the high proportion of variance ascribed to measurement error in the variance component analysis irrespectively of assessment mode. We therefore conclude that the telephone administration of the full MADRS scale is valid, conditional on the measurement precision of the scale itself.

From the results of the additional research aim, concerning item 1 (the observation item on 'apparent sadness'), we conclude that this item showed high reliability as well. Homogeneity analysis showed that item 1 fitted well into the scale. We furthermore demonstrated that for both administrations item 1 is congeneric with the 9-item scale. We therefore conclude that this item can be administered reliably by telephone.

The methodology of the present validity study seems satisfactory. The number of patients was sufficient. Furthermore, interviewers that did the second administration of the patient were not aware of the responses on the first administration. Still, the present study had some limitations.

The first limitation concerns a possible memory effect. Since interviewers were blinded, a memory effect may only occur within patients. If patients remembered how they answered the questions on the first occasion, this may have influenced their response on the second occasion. Since the MADRS was administered semi-structured, there was variation in the way the questions were formulated during each assessment. This may have diminished the memory effect within patients.

To find out whether a memory effect did exist, we assumed that the number of days between the two ratings was a proxy for the memory effect (the more time between the ratings, the less memory effect). Comparison of variance component analysis models with and without inclusion of the number of days between ratings as a covariate indicated that a memory effect could be considered limited or non-existent. Moreover, in our design it was impossible to distinguish between the memory effect and a true change in the severity of depressive symptoms (remission or regression). After all, the more days between the ratings, the more likely it was that the severity of the symptoms on the second rating differed from the first. This implies that possibly the estimates of the variance components were biased. But since we did not find much difference between estimates in models that did or did not include the number of days as a covariate, this bias seemed very limited in this case.

Second, the MADRS was originally developed as a rating scale for psychiatrists. Later, this was expanded to trained psychologists, general practitioners and nurses [26]. In the present study we used non-medically educated interviewers, who were selected on three criteria: (1) having a higher education, (2) having social skills, and (3) having an interest in the subject of depression. Our impression was that these selection criteria, in combination with our training, worked out well, though we have no data about the validity of the interviewers' ratings. However, preliminary results showed that only very little variance was due to interviewer variation, indicating that the reliability of the interviewers was high.

Third and finally, the in-person interview at the patient's home was different from the telephonic interview in several aspects. Interviewers in the face-to-face interview spent about two hours to explain the intention of the main study and to administer several scales and questionnaires, the MADRS being one of them. The telephone interview, on the other hand, took about 15 minutes and consisted solely of the administration of the MADRS. This context difference may have had an influence on the interviewer-patient relationship and on the answers patients gave. Since our results showed that the telephonic rating is as valid as the face-to-face rating, we conclude that this difference of intensity did not influence the MADRS scores.

Our overall conclusion is that the MADRS can be administered by telephone; the telephone rating of the MADRS is as valid as the usual in-person rating. The telephone administration preserves the aspect of clinical interview, can include all original items, and is less cost- and timeconsuming than a face-to-face interview. These advantages may be of interest for researchers. When choosing a depression rating scale, they may prefer the telephone administration of the MADRS to the face-to-face administration and to the MADRS-S (or any other self-rating scale).

## Competing interests

The author(s) declare that they have no competing interests.

## Authors' contributions

HPJvH conceived the idea for the study. MLMH, HJA, HvH, BT, RvD, and MdH participated in the design of the study. MLMH, HPJvH, and BT coordinated the conduct of the study and the data collection. MLMH, HJA, and HPJvH performed the statistical analyses. All authors contributed equally to the writing of this paper. All authors read and approved the final manuscript.
